# Clinical and Echocardiographic Factors of Complications in Patients With Mitral Stenosis

**DOI:** 10.7759/cureus.102266

**Published:** 2026-01-25

**Authors:** Teddy A Teddy, Edidiong Okon-Ben, Spencer Cadet, Abdullah Khan, Saad Nasir Mohmand

**Affiliations:** 1 Internal Medicine, Detroit Medical Center/Wayne State University, Detroit, USA; 2 Internal Medicine, HCA/University of Central Florida (UCF) Fort Walton Beach Hospital, Fort Walton Beach, USA; 3 Cardiology, Northwest General Hospital and Research Centre, Peshawar, PAK

**Keywords:** clinical predictors, complications, echocardiography, mitral stenosis, valvular heart disease

## Abstract

Background

Mitral stenosis (MS) is a common valvular heart disease that is frequently complicated by arrhythmias, heart failure, pulmonary hypertension, and thromboembolic events. This study aimed to determine the clinical and echocardiographic factors of complications in patients with MS.

Methodology

This retrospective cross-sectional study was conducted at the Cardiology Department of Northwest General Hospital and Research Centre, Peshawar, including 121 patients diagnosed with MS using echocardiography. Patients of either gender, aged ≥18 years, and diagnosed with MS confirmed by transthoracic echocardiography (either rheumatic or non-rheumatic) with complete clinical and echocardiographic data were included in the study. Complications were assessed at the time of initial hospital evaluation and defined as the presence of one or more disease-related adverse conditions attributable to MS, including atrial fibrillation or other clinically significant arrhythmias, heart failure, pulmonary hypertension, thromboembolic events (ischemic stroke or systemic embolism), left atrial thrombus, and respiratory complications secondary to pulmonary congestion or hypertension. Information regarding demographics, pre-existing comorbid conditions, New York Heart Association (NYHA) functional class, and multiple echocardiographic parameters was recorded. Data were analyzed using SPSS Statistics version 27 (IBM Corp., Armonk, NY, USA). Associations were assessed using chi-square and independent t-tests. Multivariate logistic regression identified independent factors. A p <0.05 was considered significant.

Results

The mean age was 57.4±16.8 years, with female predominance (63.6%). Diabetes mellitus and hypertension were present in 34.3% and 27.8% patients, respectively. Overall, 38 patients (31.4%) developed complications; arrhythmias (10.7%) and heart failure (9.3%) were most frequent, while stroke occurred in 1.7%. On univariate analysis, female sex (odds ratio (OR) 1.91, p=0.048), diabetes mellitus (OR 2.31, p=0.032), hypertension (OR 2.11, p=0.041), NYHA class III-IV (OR 3.18, p=0.003), severe MS (OR 5.38, p<0.001), pulmonary artery systolic pressure (PASP) >50 mmHg (OR 3.57, p=0.002), and left atrial diameter ≥50 mm (OR 3.44, p = 0.001) were significantly associated with complications. Multivariate analysis identified severe MS (adjusted OR (AOR) 3.58, CI 1.62-7.89, p=0.002), PASP >50 mmHg (AOR 3.12, CI 1.41-6.91, p=0.005), left atrial diameter ≥50 mm (AOR 2.74, CI 1.21-6.20, p=0.016), diabetes mellitus (AOR 2.29, CI 1.03-5.10, p=0.041), and NYHA class III-IV (AOR 2.88, CI 1.29-6.45, p=0.010) as independent factors.

Conclusion

Complications in MS are driven by both clinical and echocardiographic factors. Integrated assessment may improve risk stratification and guide timely management.

## Introduction

Valvular heart disease (VHD) includes conditions that affect heart valves and accounts for significant morbidity and mortality worldwide. Mitral stenosis (MS) is a specific type of VHD, and it's mainly caused by rheumatic fever, resulting in narrowing of the mitral valve that restricts blood flow from the left atrium to the left ventricle [1]. In developed countries, the prevalence has been decreasing due to effective prophylaxis with antibiotics and due to generally better health care. However, rheumatic heart disease is incredibly prevalent in developing parts of the world, especially in South Asia, where MS remains a prevalent clinical concern [2,3].

A study conducted in Swat, Pakistan, revealed that young adults are predominantly affected by rheumatic MS, among whom the majority are females (57.9%), and most of the patients were between 20-30 years of age. Furthermore, moderate stenosis was the commonest severity (42.1%), and isolated cases of MS were found in over two-thirds of the cases (72.4%). Importantly, nearly one-quarter of patients developed complications like arrhythmias (8.6%) and heart failure (7.9%), highlighting the ongoing clinical impact of MS in this population and the need for early identification and preventive strategies [4].

Mitral valve disease is distinguished by gradual blockage of left ventricular inflow, which results in severe hemodynamic changes such as left atrial enlargement and elevated pulmonary pressures. An increase in left atrial pressure causes dilatation of the atrium, which then becomes a substrate for atrial fibrillation and increases the risk of thromboembolic events [5-7]. Simultaneously, an increase in pulmonary pressures contributes to the development of pulmonary hypertension and deteriorates symptoms of heart failure [6,8]. Gradual ventricular dysfunction can lead to clinical heart failure as manifested by dyspnea and diminished exercise tolerance [9]. There is no difference in the risk of thromboembolic events among different categories of mitral regurgitation severity as opposed to the traditional view that mitral regurgitation decreases thrombus formation [10]. Some patients may remain asymptomatic despite these pathophysiological changes for many years, thus there is a need for early diagnosis and vigilant monitoring [5].

Echocardiography plays a pivotal role in the diagnosis and management of MS, offering extensive assessment of mitral valve anatomy, function and associated cardiac structural alterations. Both transthoracic and transesophageal echocardiography allow for great details in valve morphology and pathology, hence guiding clinical decision-making andtherapeutic planning [11]. The severity of the disease is conventionally assessed by echocardiographic parameters such as the area of the mitral valve and transmitral pressure gradients, which have shown close association with clinical events [8,12]. Disease progression is further evaluated by echocardiography by measuring left atrial size, volume, and the motion abnormalities of the valve, all carrying important prognostic implications [12,13]. Standardized scoring systems, particularly the Wilkins score, enhance the value of echocardiography by quantifying valvular deformity and supporting risk stratification and interventional strategies [13].

Even with similar areas of the mitral valve, clinical outcomes can diverge, influenced by a combination of clinical and echocardiographic factors that are at the root of the progression of the disease and prognosis. Atrial fibrillation is a powerful determinant of poor outcome, mainly in patients with low-gradient MS [14], but other comorbidities such as hypertension, diabetes, and chronic kidney disease also substantially increase mortality [15]. Echocardiography adds to this: a low left ventricular ejection fraction - particularly less than 40% - and higher pulmonary artery systolic pressure (PASP) are reliable predictors of poor outcome [15,16]. More symptomatic status, represented by higher New York Heart Association (NYHA) class, is associated with increased mortality and heart failure burden [16]. Even in patients with similar anatomical severity, results differ, highlighting individual risk profiling and personalized management.

The integration of clinical and echocardiographic variables is essential for comprehensive assessment of patients with MS, as anatomical severity alone does not fully explain the wide variability in clinical outcomes. Although regional studies have reported the prevalence and clinical characteristics of MS, there remains limited evidence integrating clinical factors with echocardiographic parameters to identify independent predictors of complications at the time of presentation. Also, limited data exist on the clinical and echocardiographic predictors of complications in patients presenting at older ages or with mixed rheumatic and degenerative etiologies. Clinical variables such as symptom burden and comorbid conditions interact with echocardiographic measures of structural remodeling and hemodynamic severity to influence adverse outcomes. Therefore, the present study aims to evaluate combined clinical and echocardiographic predictors of complications in patients with MS using a single multivariable model, with the objective of improving risk stratification, enabling early identification of high-risk patients, and supporting timely, individualized management.

## Materials and methods

This retrospective cross-sectional study was conducted at the Cardiology Department of Northwest General Hospital and Research Centre, including 121 adult patients diagnosed with MS. The sample size was calculated through OpenEpi software keeping the anticipated frequency of arrhythmias among MS patients as 8.6% [4], confidence level of 95% and 5% margin of error. The inclusion criteria were patients of either gender, aged ≥18 years, diagnosed with MS confirmed by transthoracic echocardiography (either rheumatic or non-rheumatic) and availability of complete clinical and echocardiographic data. Patients with significant concomitant valvular lesions requiring immediate surgical intervention, a history of prior mitral valve surgery or percutaneous mitral intervention, congenital mitral valve disease, or incomplete clinical or echocardiographic records were excluded.

Data collection was started after taking prior permission from the hospital IRB committee under Ref No: NwGH/MPED/6373; dated 1st June 2025, from the hospital electronic record system from January 2024 to May 2025, using a structured proforma. The information regarding age, gender, duration of symptoms, NYHA functional class, atrial fibrillation presence, thromboembolic events history, comorbidities (hypertension, diabetes), echocardiographic variables (like mitral valve area, mean transmitral gradient, left atrial size, presence of left atrial thrombus, PASP, and severity of MS) and complications was recorded.

Etiology of MS (rheumatic or non-rheumatic) was recorded when explicitly documented in echocardiography reports or clinical records. However, detailed etiologic classification was not available for all patients and was analyzed descriptively.

Complications were defined as disease-related adverse conditions attributable to MS, including atrial fibrillation or other clinically significant arrhythmias, heart failure, pulmonary hypertension, thromboembolic events (ischemic stroke or systemic embolism), left atrial thrombus, and respiratory complications secondary to pulmonary congestion or hypertension. Although patients could have multiple complications, only the primary complication documented at presentation was recorded for analysis. Primary complication was defined as the complication most directly attributable to MS or deemed most clinically significant based on hospital records. Complications were assessed at the time of initial hospital evaluation based on clinical findings, ECG, and echocardiography. Baseline comorbid conditions (like diabetes mellitus and hypertension) were recorded separately and were not considered complications. Diabetes mellitus and hypertension were defined based on a documented prior diagnosis in the medical record or current use of antidiabetic or antihypertensive medications at presentation. 

MS was defined as the echocardiographically confirmed constriction of the mitral valve that leads to the limitation of the diastolic blood flow from the left atrium to the left ventricle. Mitral valve area by two-dimensional planimetry and/or pressure half-time method was used to determine the severity of MS (which was mild (>1.5 cm 2), moderate (1.0-1.5 cm 2), or severe (<1.0 cm 2)). In the case of its presence, mitral regurgitation has been independently graded as mild, moderate, and severe on the basis of standard echocardiographic criteria [17]. Mitral regurgitation severity was reported descriptively only, and all echocardiographic severity measures were based on baseline assessment at presentation.

Valve involvement was categorized as isolated mitral valve disease and isolated non-mitral valve disease (aortic, tricuspid, or pulmonic). Involvement was defined by the echocardiographic presence of stenosis and/or regurgitation and was reported descriptively without severity stratification. 

Pulmonary hypertension was defined as a PASP >50 mmHg on Doppler echocardiography, consistent with moderate-to-severe pulmonary hypertension as described in standard echocardiographic guidelines. PASP was estimated using the tricuspid regurgitation velocity on Doppler echocardiography. Left atrial enlargement was defined as a left atrial diameter ≥50 mm on echocardiography. Left ventricular systolic function was measured using the Simpson biplane method, with a systolic function <40% considered reduced [18].

The identification of atrial fibrillation was predisposed on the basis of electrocardiographic records of irregularly irregular rhythm with no recognizable P waves or a prior known diagnosis [19]. Heart failure was characterized by typical clinical symptoms and functional restriction and categorised based on the NYHA functional classification, with advanced symptoms being defined by NYHA class III-IV. The thromboembolic events were categorized as described ischemic stroke or systemic embolism that were confirmed clinically and/or through imaging [20]. 

Data was analysed through SPSS Statistics version 27 (IBM Corp., Armonk, NY, USA). Continuous variables like age were expressed as mean ± standard deviation after checking normality through Shapiro Wilk test. Age was analyzed as a continuous variable for inferential analysis. For descriptive purposes, age was additionally categorized into groups based on the observed data distribution to facilitate clinical interpretation. Categorical variables like gender, comorbidities, complications, valvular involvement, and severity grading of mitral valve disease were presented as frequencies and percentages. Factors associated with complications at presentation were assessed using chi-square tests and independent t-tests as appropriate. Multivariate logistic regression (MLR) analysis was performed to identify independent factors of complications. Variables in MLR were selected based on clinical relevance and univariate analysis with a p-value <0.10. Model goodness-of-fit was assessed using the Hosmer-Lemeshow test, and discriminative ability was evaluated by the area under the receiver operating characteristic (ROC) curve. Multicollinearity among echocardiographic variables was examined using variance inflation factors (VIF), with no evidence of significant collinearity observed. The final model maintained an acceptable events-per-variable ratio to ensure model stability. A p-value of <0.05 was considered statistically significant. 

## Results

This study includes 121 patients diagnosed with MS. The mean age of patients was 57.4±16.8 years, with a female predominance (77, 63.6%). Age was additionally categorized into groups based on the observed data distribution. Most of the patients were above 60 years old. Diabetes mellitus and hypertension were present in 41 (34.3%) and 34 (27.8%) patients, respectively, and were defined based on a documented prior diagnosis in the medical record or current use of antidiabetic or antihypertensive medications at presentation (Table 1). 

**Table 1:** Demographic and Clinical Characteristics (N = 121)

Variable	Category	n (%)
Gender	Male	44 (36.4%)
	Female	77 (63.6%)
Age group (years)	20–40 years	33 (27.3%)
	40–60 years	13 (10.8%)
	> 60 years	75 (61.9%)
Diabetes mellitus	Present	41 (34.3%)
Hypertension	Present	34 (27.8%)

Rheumatic MS predominantly affected younger patients and females, whereas non-rheumatic MS occurred at an older age and was more frequently associated with chest pain, with these differences being statistically significant. In contrast, no significant differences were observed between the two groups in terms of advanced functional limitation (NYHA class III-IV), severity of MS, pulmonary hypertension, left atrial enlargement, or overall complications at presentation (Table 2).

**Table 2:** Clinical and Echocardiographic Characteristics According to Etiology of Mitral Stenosis (MS) (N = 121) ¹ Symptom severity was assessed using the New York Heart Association (NYHA) functional classification [20]. ² Echocardiographic grading of mitral stenosis severity, pulmonary artery systolic pressure (PASP) estimation, and left atrial diameter measurement were performed using standard transthoracic echocardiographic criteria [12,17,18].

Variable	Rheumatic MS (n = 82)	Non-rheumatic MS (n = 39)	p-value
Mean age (years)	48.5 ± 13.3	66.7 ± 11.2	<0.001
Female sex	58 (70.7%)	19 (48.7%)	0.021
Chest pain	26 (31.7%)	23 (59.0%)	0.004
NYHA class III–IV^1^	34 (41.5%)	19 (48.7%)	0.462
Severe mitral stenosis^2^	15 (18.3%)	11 (28.2%)	0.197
PASP >50 mmHg^2^	24 (29.3%)	13 (33.3%)	0.653
Left atrial diameter ≥50 mm^2^	27 (32.9%)	16 (41.0%)	0.377
Any complication at presentation	22 (26.8%)	16 (41.0%)	0.096

**Figure 1:**
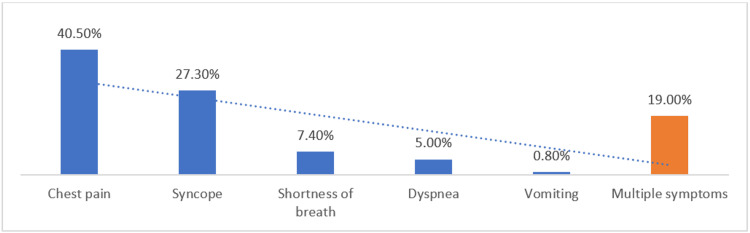
Presenting Symptoms Among Patients With Mitral Stenosis (N = 121)

Chest pain (40.5%) and syncope (27.3%) were the most frequently reported presenting symptoms, while dyspnea and shortness of breath were less commonly documented. About 19% of patients presented with multiple symptoms (Figure 1).

Overall, 38 of 121 (31.4%) patients developed at least one complication. Only the primary complication documented at presentation was recorded for each patient. Arrhythmias (13/121, 10.7%) and heart failure (11/121, 9.3%) were the most frequent complications, while stroke occurred in two of 121 (1.7%) patients (Table 3).

**Table 3:** Complications Among Patients With Mitral Stenosis (N = 121)

Complication	n (%)
No complication	83 (68.6%)
Any complication	38 (31.4%)
Arrhythmia	13 (10.7%)
Heart failure	11 (9.3%)
Stroke	2 (1.7%)
Respiratory complications	12 (9.9%)

Valve involvement was categorized as isolated mitral valve disease and isolated non-mitral valve disease (aortic, tricuspid, or pulmonic). Involvement was defined by the echocardiographic presence of stenosis and/or regurgitation and was reported descriptively. The mitral valve was the most frequently affected valve (84, 69.4%), followed by tricuspid (21, 17.4%), aortic (11, 9.1%), and pulmonic valves (five, 4.1%) (Table 4). 

**Table 4:** Valvular Involvement Among Patients With Mitral Stenosis (N = 121)

Valve involved	n (%)
Mitral valve	84 (69.4%)
Tricuspid valve	21 (17.4%)
Aortic valve	11 (9.1%)
Pulmonic valve	5 (4.1%)

Moderate MS was the most prevalent severity grade (51, 42.1%), followed by mild (44, 36.4%) and severe (26, 21.5%). Concomitant mitral regurgitation was observed in a subset of patients with MS (mild in 57%, moderate in 27.3%, and severe in 15.7%), and severity categories for MS and mitral regurgitation were not mutually exclusive (Table 5).

**Table 5:** Severity Grading of Mitral Valve Disease (N = 121) Severity of mitral stenosis and mitral regurgitation was graded using standard transthoracic echocardiographic criteria based on mitral valve area and regurgitation severity parameters [17].

Disease	Severity	n (%)
Mitral stenosis	Mild	44 (36.4%)
	Moderate	51 (42.1%)
	Severe	26 (21.5%)
Mitral regurgitation	Mild	69 (57.0%)
	Moderate	33 (27.3%)
	Severe	19 (15.7%)

Female patients, those with diabetes mellitus, and hypertension had higher odds of complications (OR 1.91, 2.31, and 2.11, respectively; p < 0.05). Patients with advanced symptoms (NYHA class III-IV) were more likely to develop complications (OR 3.18, p = 0.003). Echocardiographic parameters including severe MS, PASP >50 mmHg, and left atrial diameter ≥50 mm were strongly associated with complications (OR 5.38, 3.57, and 3.44, respectively; p ≤ 0.002). Patients who developed complications were significantly older than those without complications (mean age: 59.5 ± 6.8 years vs. 39.8 ± 7.6 years, respectively; p = 0.047). This difference reflects the concentration of complications among older patients, whereas younger patients predominantly presented without complications (Table 6).

**Table 6:** Factors Associated With Complications at Presentation (N = 121) ¹ Symptom severity was assessed using the New York Heart Association (NYHA) functional classification [20]. ² Echocardiographic grading of mitral stenosis severity, pulmonary artery systolic pressure (PASP) estimation, and left atrial diameter measurement were performed using standard transthoracic echocardiographic criteria [12,17,18].

Predictor Type	Variable	Complication Present n (%)	No Complication n (%)	p-value
Clinical	Female sex	28 (73.7%)	49 (59.0%)	0.048
	Diabetes mellitus	18 (47.4%)	23 (27.7%)	0.032
	Hypertension	15 (39.5%)	19 (22.9%)	0.041
	NYHA class III–IV¹	24 (63.2%)	29 (34.9%)	0.003
Echocardiographic^2^	Severe mitral stenosis	16 (42.1%)	10 (12.0%)	<0.00
	PASP >50 mmHg	19 (50.0%)	18 (21.7%)	0.002
	Left atrial diameter ≥50 mm	21 (55.3%)	22 (26.5%)	0.001
Demographic	Mean age (years)	59.5± 6.8	39.8 ± 7.6	0.047

On multivariate analysis, severe MS, PASP >50 mmHg, left atrial diameter ≥50 mm, diabetes mellitus, and NYHA class III-IV were independent factors of complications in patients with MS (p ≤ 0.041). Patients with these characteristics had 2.3-3.6 times higher odds of developing complications, whereas female gender was not independently associated with risk (p = 0.337). Adjusted odds ratios (OR) with 95% confidence intervals (CI) for independent factors are presented in Table 7.

**Table 7:** Multivariate Logistic Regression Analysis for Factors Associated With Complications at Presentation ¹ Functional status was assessed using the New York Heart Association (NYHA) functional classification [20]. ² Echocardiographic parameters including mitral stenosis severity, pulmonary artery systolic pressure (PASP), and left atrial diameter were defined and graded according to standard echocardiographic criteria [12,17,18]. * Model goodness-of-fit: Hosmer–Lemeshow p = 0.67; model discrimination: AUC = 0.82. * Variables with p < 0.10 on univariate analysis and those deemed clinically relevant were included in the multivariate model. * Multicollinearity among echocardiographic variables was assessed using variance inflation factor (VIF); all included variables had VIF < 2, indicating no significant collinearity

Variable	Adjusted OR	95% CI	p-value
Severe mitral stenosis^2^	3.58	1.62–7.89	0.002
PASP >50 mmHg^2^	3.12	1.41–6.91	0.005
Left atrial diameter ≥50 mm^2^	2.74	1.21–6.20	0.016
Diabetes mellitus	2.29	1.03–5.10	0.041
NYHA class III–IV^1^	2.88	1.29–6.45	0.010

## Discussion

This study identified the clinical and echocardiographic factors associated with complications at presentation in patients with MS and demonstrated that adverse outcomes were the result of a complex interplay of disease severity, cardiac remodeling, symptom burden, and co-morbid conditions. The observed rates of complications in our study are broadly comparable to those reported in regional studies when differences in study design and outcome definitions are considered. The mean age of patients in our cohort compares with reports from middle-aged to elderly populations described by Shabeer (2025) [21] and Batista et al. (2024) [22] and reflects the dual burden of rheumatic and degenerative MS. Although rheumatic MS traditionally affects younger populations, the relatively higher mean age observed in our cohort likely reflects a mixed population with both rheumatic and degenerative MS, as well as delayed presentation inherent to hospital-based retrospective studies.

The female predominance reported in this study (63.6%) also falls in line with the results of multiple regional and international studies reporting 62 to 78%, hence confirming that MS disproportionately burdens women. However multivariate analysis demostrates that the outcomes would be influenced more strongly by disease severity and co-morbidities rather than gender alone, as noted by Santos-Alfaro et al. (2025) [23].

In our study, diabetes mellitus presents itself at 34.3% and hypertension at 27.8%, in line with literature citing an increasing prevalence of metabolic and cardiovascular comorbidities in multiple sclerosis, particularly among the elderly. Diabetes is clearly an independent risk factor for complications, confirming studies reported by Santos-Alfaro et al. (2025) [23] and Leow et al. (2024) [24], who associate diabetes with poor cardiovascular outcomes such as heart failure and increased mortality. Diabetes mellitus may worsen outcomes in MS through endothelial dysfunction, accelerated myocardial fibrosis, and impaired diastolic relaxation, thereby increasing susceptibility to heart failure and arrhythmias

Higher symptom burden, specifically NYHA class III-IV, also correlates independently with complications in our group. This is essentially in line with Shabeer (2025) [21], who mentioned that many patients already have substantial functional limits at the time of presentation. The severity of symptoms has remained a solid marker of hemodynamic strain and disease progression, consistently associated with poorer outcomes such as heart failure and more hospitalizations. Although chest pain is not a classical presenting feature of isolated MS, its predominance in this cohort may be explained by severe pulmonary hypertension, right ventricular strain, atrial ischemia, or coexistent coronary artery disease, particularly in older patients. Similar observations have been reported in hospital-based cohorts with advanced disease and mixed degenerative and rheumatic etiologies

Echocardiographic severity was the most powerful determinant of complications in this study. Severe MS independently enhanced the risk of adverse outcomes, consistent with previous reports showing that a mitral valve area below 1.0 cm² is associated with increased morbidity. Although Batista et al. (2024) [22] reported a lower proportion of severe disease, our higher prevalence likely reflects delayed presentation and limited access to early intervention in resource-limited settings. However, comparisons with prior studies should be interpreted in light of differences in study design, population characteristics, and duration of follow-up, as many published cohorts include younger patients with predominantly rheumatic disease and prospective longitudinal assessment. 

The other factor that showed an association with complications was elevated PASP >50 mmHg. This corresponds to the findings by Leow et al. (2024) [24] and Batista et al. (2024) [22], which showed that pulmonary hypertension is associated with a significantly worse prognosis due to an increased risk of heart failure, arrhythmias, and mortality. Pulmonary hypertension is often an advanced stage of disease and limits therapeutic options, so early identification is necessary. Elevated PASP reflects chronic pressure overload and pulmonary vascular remodeling, which contributes to right ventricular dysfunction, worsening symptoms, and increased risk of adverse clinical events.

Left atrial enlargement (≥50 mm) was independently associated with complications, corroborating prior studies that relate atrial dilation with the development of atrial fibrillation, thrombus formation, and systemic embolization. Khan et al. (2025) [25] and Santos-Alfaro et al. (2025) [24] also similarly reported a strong relationship between left atrial size and adverse outcomes, emphasizing the prognostic value of atrial remodeling beyond mitral valve area alone.

Among the clinically overt complications reported, arrhythmias, predominantly atrial fibrillation, were the most frequently observed. This is consistent with literature reporting a prevalence as high as 63% in MS patients. The prevalence in our cohort was lower than that reported in long-term rheumatic MS cohorts; however, the observed association with left atrial enlargement supports prior evidence that atrial remodeling constitutes a key arrhythmogenic substrate. The reliance on documented ECG findings rather than continuous rhythm monitoring may also have contributed to the underdetection of paroxysmal atrial fibrillation. Heart failure was present in almost 9% of patients, similar to long-term follow-up data from Pascual et al. (2025) [26], and was strongly related to pulmonary hypertension and advanced symptoms.

The incidence of stroke was generally low within our cohort, at 1.7%, and may reflect either underdiagnosis or good anticoagulation in selected patients, but long-term stroke rates have been reported to be higher by other groups, particularly among patients with atrial fibrillation, reinforcing the importance of vigilant thromboembolic risk assessment. Respiratory complications were also observed and are likely secondary to pulmonary congestion and elevated pulmonary pressures associated with advanced MS. Similar associations have been described in patients with longstanding disease and pulmonary hypertension, underscoring the systemic impact of delayed presentation [23, 27]. 

The proposed integration of clinical and echocardiographic factors into routine assessment is inferred from the observed associations in this study and supported by existing literature, rather than being directly tested as an interventional strategy. These findings are most applicable to similar hospital-based settings in resource-limited regions where patients often present at advanced stages of MS and access to longitudinal follow-up may be limited. The findings may assist in clinical profiling and identification of patients with a higher burden of complications at the time of presentation

Study limitations and future implications

The study limitation includes its single-center nature and retrospective design with small sample size, which may introduce selection bias and limit generalizability. Identified factors represent associations rather than causal relationships, and treatment-related variables such as anticoagulation use and prior interventions were not consistently available, representing potential unmeasured confounders. The absence of longitudinal follow-up further restricts causal inference. As complications and exposures were assessed simultaneously, temporal relationships could not be established. Prospective, multicenter studies with long-term follow-up are needed to validate these factors, assess outcomes including atrial fibrillation, thromboembolic events, heart failure hospitalization, and valve interventions, and refine the prognostic value of combined clinical and echocardiographic risk models.

## Conclusions

In this retrospective cross-sectional study, complications in patients with MS were found to be associated with increased disease severity, elevated pulmonary artery pressures, left atrial enlargement, advanced functional class, and diabetes mellitus at presentation. These associations highlight the importance of comprehensive clinical and echocardiographic assessment in patients presenting with MS. Causal inferences cannot be drawn, and prospective studies are required to validate these observations.
